# Functional Interplay between Small Non-Coding RNAs and RNA Modification in the Brain

**DOI:** 10.3390/ncrna4020015

**Published:** 2018-06-07

**Authors:** Laura J. Leighton, Timothy W. Bredy

**Affiliations:** Cognitive Neuroepigenetics Laboratory, Queensland Brain Institute, The University of Queensland, Brisbane, QLD 4072, Australia; t.bredy@uq.edu.au

**Keywords:** epitranscriptomics, neurobiology, non-coding RNA, small RNA

## Abstract

Small non-coding RNAs are essential for transcription, translation and gene regulation in all cell types, but are particularly important in neurons, with known roles in neurodevelopment, neuroplasticity and neurological disease. Many small non-coding RNAs are directly involved in the post-transcriptional modification of other RNA species, while others are themselves substrates for modification, or are functionally modulated by modification of their target RNAs. In this review, we explore the known and potential functions of several distinct classes of small non-coding RNAs in the mammalian brain, focusing on the newly recognised interplay between the epitranscriptome and the activity of small RNAs. We discuss the potential for this relationship to influence the spatial and temporal dynamics of gene activation in the brain, and predict that further research in the field of epitranscriptomics will identify interactions between small RNAs and RNA modifications which are essential for higher order brain functions such as learning and memory.

## 1. Introduction

Cells produce vast amounts of RNA which lack protein-coding potential, including many functional RNA molecules less than 200 nucleotides in size. These small non-coding RNAs arise through a multitude of distinct biogenesis pathways, and are involved in cellular processes including transcription, splicing, translation, RNA modification, and regulation of gene expression through several mechanisms (Table 1). Some small RNAs (for instance, transfer RNAs or tRNAs) participate in the most basic processes of life and are conserved from prokaryotes to humans, and expressed in every living cell at every developmental stage. Others show extraordinary precision and specificity of expression, with their existence restricted to particular cells or cell lineages, or occurring transiently in response to environmental cues. In addition, new roles are being identified for fragmented or modified forms of constitutively expressed small RNAs, which were previously disregarded as artefacts of sample preparation and sequencing, but are now known to be functional in their own right. 

Small non-coding RNAs have important regulatory roles in all mammalian cell types, but seem to be particularly important in the brain [1,2]. Higher order brain functions, including learning and memory, are underpinned by rapid activity-dependent changes in gene expression and protein synthesis [3,4]. Small regulatory RNAs (particularly microRNAs or miRNAs) which repress messenger RNA (mRNA) expression at the post-transcriptional level provide a rapid and adaptive means for modulation of gene expression, which is ideally suited to accommodate the regulatory demands of neurons. Other small regulatory RNAs (particularly piwi-interacting RNAs or piRNAs) are able to modulate gene expression at the epigenetic level, and are therefore likely to be involved in establishing and maintaining long-term patterns of RNA expression. Regulation of gene expression is also emerging as a surprising mechanism of action for small RNAs with functions that were believed to be entirely unrelated; for example, transfer RNA fragments have been implicated in stress responses and in transgenerational epigenetic inheritance [5], while small nuclear RNAs (snRNAs) can influence alternative splicing [6].

Post-transcriptional modifications can profoundly influence the structure, stability and base-pairing properties of RNA, and are increasingly recognised as a mechanism for regulating RNA localisation, longevity, translation, and interactions with other molecules [7,8]. Several classes of small non-coding RNA, notably small nuclear RNAs and transfer RNAs, undergo extensive epitranscriptomic modification. Recent studies have uncovered proteins which read, write and erase RNA modifications predominantly on small non-coding RNAs, and have found that mutations affecting these proteins are disproportionately damaging to the brain [9]. Other small RNAs function by providing sequence specificity to proteins which apply RNA modifications, in order to guide them to the correct location on target RNA molecules. For example, small RNA-guided modifications are essential for the function of the spliceosome and the ribosome and may play a role in translational regulation [10,11]. Finally, epitranscriptomic marks occurring on mRNA can influence its interaction with small non-coding RNAs by creating or ablating small RNA binding sites and may even affect translation dynamics or the sequence of proteins by affecting base pairing between mRNA and the transfer RNA anticodon loop during translation [12].

In this review, we explore several classes of small non-coding RNAs which are known to be expressed in the mammalian brain, discuss their roles in brain function, and consider how post-transcriptional modifications of small RNAs and their targets can increase the information-carrying capacity of neuronal RNA. We predict that as new technology permits the profiling and functional interrogation of the epitranscriptome with improved temporal and spatial resolution, new mechanisms of gene regulation and cellular signalling will be identified at the interface of small RNA biology and RNA modification and shown to be essential for higher-order neurological function, including learning and memory. 

## 2. Small RNAs

### 2.1. MicroRNAs

MicroRNAs are a class of small regulatory RNAs which negatively regulate gene expression through interactions with mRNA. Mature microRNAs are incorporated into a ribonucleoprotein known as the RISC (RNA-induced silencing complex), which is guided to specific sites by the base-pairing of part of the miRNA with complementary regions in the 3′ untranslated region (UTR) of mRNAs [13,14]. Depending on the degree of sequence complementarity, this can result in degradation of the mRNA or impairment of its translation [13,15]. The rapid kinetics of miRNA-mediated regulation make this process particularly important in neurons, which must adapt to constantly-changing inputs on a faster timescale than any other cell type. Concomitantly, the mammalian brain expresses a large diversity of miRNAs compared to most other tissue types [16,17,18,19,20].

#### 2.1.1. Biogenesis of MicroRNAs

In mammals, the majority of miRNAs are derived from hairpin-containing precursor RNAs (pri-miRs) which are transcribed by RNA polymerase II. These precursors are processed in the nucleus by the endonuclease DROSHA which trims the RNA to a single hairpin (pre-miR) which is exported from the nucleus. Final miRNA processing and maturation occurs in the cytoplasm, where the endonuclease DICER removes the loop region from the hairpin, with several enzymes then participating in strand selection and loading of the miRNA into the RISC (reviewed by Mott et al. [13], Kim et al. [21] and Bartel et al. [22]).

A small percentage of miRNAs are derived from alternative precursors which bypass the requirement for DROSHA. The best-studied examples are mirtrons, which are whole introns of protein-coding genes that act as miRNA precursors. After their removal from the host transcript by splicing, they are exported from the nucleus and processed by DICER [23]. Rarely, miRNAs may be derived from other small RNAs, including snRNAs, small nucleolar RNAs (snoRNAs) and transfer RNAs ([15,24], discussed elsewhere in this review).

Although the first steps of miRNA biogenesis canonically occur in the nucleus, it is important to note that miRNA biogenesis in neurons can occur at synapses in a locally regulated manner. Pri-miRs, pre-miRs and the miRNA biogenesis machinery (including DROSHA, DGCR8 and DICER) occur in synapses of the adult mouse brain, and many miRNAs are strongly enriched in the synaptic compartment compared to whole cell lysate [25,26]. It is believed that miRNA precursors are trafficked to synapses in a similar manner to mRNAs, and there is evidence that structural features of pri-miRs play a role in determining which pri-miRs are selected for synaptic localisation [26,27]. This ability to rapidly produce specific miRNAs at the synapse in response to synaptic activity supports the hypothesis that miRNAs are essential components of localised gene regulatory networks at the synapse and are involved in the regulation of neuronal plasticity.

#### 2.1.2. Neuronal MicroRNAs Fine-Tune Gene Expression

Mammals have over one thousand miRNA sequences, almost all of which target dozens or hundreds of genes [14,15,22]. Many miRNAs occur in families with similar sequences and many shared targets between family members, and genomic deletion of individual miRNAs often does not produce an obvious phenotype [15]. The 3′ UTRs of most mRNAs also contain binding sites for multiple miRNAs [13,14]. These features have led to the hypothesis that miRNAs operate as part of large gene regulatory networks, acting as molecular buffers to constrain damaging fluctuations in protein expression in different subcompartments of the cell [15]. This is supported by the observation that global miRNA expression is regulated in neurons in response to activity, as well as the finding that widespread changes in miRNA expression in the brain co-occur with neurological disorders and perturbations, including induced ischemia and seizures [28], Alzheimer’s disease [29] and amyotrophic lateral sclerosis [30].

However, there are also many examples of individual miRNAs which have profound impacts on neuronal function in isolation, particularly in the context of learning and memory. For instance, miR-132 is regulated by the neuronal transcription factor CREB (cAMP response element binding factor); this miRNA is upregulated in the mouse hippocampus in response to a spatial memory task, and mild overexpression of this miRNA enhances cognitive performance [31]. MiR-182 is downregulated in the rat amygdala by auditory fear conditioning and its overexpression disrupts long-term memory [32], while miR-144-3p is upregulated in the mouse amygdala by fear extinction learning and targets multiple genes involved in plasticity-associated signalling [33]. Some plasticity-related miRNAs are enriched in the brain; for instance, miR-128b, which regulates the formation of fear extinction memory in the mouse prefrontal cortex, is highly expressed in the brain [34]. These and several other examples have been thoroughly reviewed elsewhere [1,15,35]. It is noteworthy that many known functions of neuronal miRNAs occur at the synapse (reviewed by Hu et al. [36]). Synapses may be located many centimetres from the neuronal nucleus, and this physical distance creates the need for an autonomous localised network of gene regulation at the synapse. Messenger RNAs and the protein synthesis machinery are trafficked to synapses, where translation occurs in response to neuronal activity and is required for synaptic plasticity [37]. The contribution of miRNAs to the regulation of local protein synthesis at synapses bypasses the need to signal back to the nucleus, thereby uniquely meeting the need of the neuron to respond to synaptic activity in real time.

#### 2.1.3. Intercellular Transfer of MicroRNAs

There is a growing recognition that miRNAs are commonly packaged into extracellular vesicles as a means of communication between cells [38]. Much research has focused on the presence of miRNA-containing exosomes in blood, particularly with regard to their value as biomarkers in the context of cancer and other diseases. However, miRNA-containing exosomes can also permit cell–cell communication over short distances, including within the central nervous system (CNS), and the miRNA content of brain exosomes can be altered by schizophrenia and bipolar disorder [39], glioblastoma [40] and prion disease [41]. Neurons release exosomes containing miRNAs as a means to communicate with glia. For instance, miR-124a-containing exosomes released by neurons have been shown to modulate expression of the astroglial glutamate transporter in vitro and in vivo [42]. Similarly, neural stem cells in the mouse neonatal subventricular zone release exosomes which contain multiple miRNAs that modulate the morphology and function of microglia [43]. Glia also release exosomes which have been shown to transfer miRNAs to neurons; miR-146a-5p, which is only produced in inflammatory microglia, is transferred to hippocampal neurons and decreases the number and strength of excitatory synapses [44]. Exosomal transfer of miR-133b from mesenchymal stromal cells to neurons also occurs and is important during recovery from brain injury as a mechanism by which neurite outgrowth is regulated [45]. Finally, release of miRNA-containing exosomes by neurons is inducible by neuronal activity [46]. The miRNAs contained in these exosomes are thought to communicate in neuron–neuron communication [47,48] but miRNA exocytosis may also be a mechanism to rapidly deplete miRNAs from the presynaptic compartment [46]. Future research will improve our understanding of how miRNAs are selected for incorporation into exosomes under various conditions, and how this means of cell-cell communication works within the nervous system.

#### 2.1.4. MicroRNAs and Their Precursors Can Be Modified

Recent research has identified the presence of modified bases in miRNA precursors and mature miRNAs. Several papers have focused on N6-methyladenosine (m6A), which is often considered a functional switch on RNA. Yuan et al. showed that methylation of miR-125b in HeLa cells inhibits the function of this miRNA at all three stages of its biogenesis; the presence of m6A inhibits the processing of pri-miR-125b2 into pre-miR-125b2 by Drosha, decreases the cleavage of this pre-miR by Dicer, and attenuates incorporation of the mature miRNA into the RISC [49]. However, Alarcón et al. found that m6A in MDA-MB-231 cells is targeted to pri-miRNAs by Mettl3 and marks them for recognition and processing; knockdown of Mettl3 globally decreases mature miRNA levels while causing unprocessed pri-miRNAs to accumulate, strongly implicating m6A more generally in miRNA biogenesis [50]. This is supported by the finding that the m6A reader protein HNRNPA2B1 interacts with the Drosha cofactor DGCR8 to facilitate pri-miRNA processing [51]. This RNA modification also occurs on mature miRNAs; Berulava et al. showed that over 200 miRNAs from HEK293 cells are immunoprecipitated by an antibody against m6A, and that knockdown of the m6A demethylase fat mass and obesity associated protein (FTO) alters the expression levels of several dozen [52]. Another study reported that the formation of 8-hydroxyguanosine (8-OHG) within miR-184 occurs as a result of exposure to reactive oxygen species and that this enables miR-184 to target Bcl-xL and Bcl-W, with which it does not otherwise interact [53]. Suppression of these genes by modified miR-184 promotes apoptosis as a response to oxidative stress in rat heart cells. To our knowledge, there is currently no direct evidence for base-modified miRNAs in the brain, but we predict that further research will confirm that these mechanisms exist in neurons and contribute to the cell-type and state-dependent selectivity of miRNA action underlying plasticity, learning and memory.

MicroRNAs can also be modified by the addition of non-templated nucleotides to the 3′ end, which can occur at the miRNA precursor stage as well as to mature miRNAs [54,55]. MicroRNAs with sequences differing at the 3′ end through this process are termed isomiRs. Given that the targeting region of the miRNA is at the 5′ end, the addition of nucleotides at the 3′ end is likely a means to regulate miRNA processing or turnover rather than affecting targeting by directly altering base-pairing with the miRNA, and this is supported by the existing evidence. Oligouridylation of the 3′ end of pre-miRs inhibits their recognition by Dicer and reduces accumulation of the mature miRNA; however, monouridylation enhances Dicer cleavage and increases the quantity of mature miRNA produced [54,56,57]. The addition of non-templated cytosine and adenine to pre-miRs and mature miRNAs is also observed [54,55]; 3′ adenylation of miR-122 enhances its stability [58], whereas the same modification triggers degradation of miR-21 [59]. Another study found that 3′ adenylation reduces the effectiveness of mRNA targeting, possibly by impairing incorporation of the miRNA into the RISC [60]. Non-templated nucleotide addition is known to occur in the mouse hippocampus, with around 20% of miRNA reads containing either one or two non-templated nucleotides [55], implying that this method of regulating miRNA function is employed by neurons.

Another form of miRNA modification is adenosine-to-inosine (A-to-I) RNA editing catalysed by enzymes of the ADAR (adenosine deaminase acting on RNA) family, which target double-stranded RNA templates including miRNA precursors. Adenosine-to-inosine RNA editing profoundly changes the base-pairing properties of the RNA molecule; adenosine base-pairs with uridine, whereas inosine preferentially pairs with cytidine but can also pair with adenosine or uridine. This can cause mature miRNAs containing inosine to target different mRNAs from their unedited counterparts [61], and A-to-I editing events are enriched in the seed region of human miRNAs [62]. Adenosine-to-inosine editing of miRNAs occurs at a higher level in the brain than in other human tissue types [62], and impairment of this process is associated with glioblastoma [62,63]. Adenosine-to-inosine editing of miRNA precursors also regulates miRNA biogenesis by destabilising the pri-miRNA structure, thereby impairing pri-miRNA recognition and processing by DROSHA [64].

#### 2.1.5. MicroRNAs May Be Functionally Modulated by mRNA Editing and RNA Modification

Modification and editing of mRNAs could affect their potential to be regulated by miRNAs by creating or ablating miRNA binding sites, or by affecting the structure of the mRNA molecule in a way that makes it more or less accessible to the RISC. Editing of A-to-I by ADAR enzymes occurs in mRNA, particularly in the brain [65]. Inosine can pair with adenosine, uridine or cytidine, and its presence in human mRNA has been shown to generate novel miRNA binding sites [66]. Similarly, cytidine deamination by the APOBEC (apolipoprotein B mRNA editing enzyme, catalytic polypeptide-like) enzyme family converts cytidine residues to uridines, which also has the potential to create or ablate binding sites for miRNAs by effectively changing the mRNA sequence. Sites of APOBEC editing are most common in the 3′ UTR on of mouse mRNAs, and more than 35% occur within sequences matching known miRNA targets, which would likely be ablated in those transcripts which are edited [67]. It is also plausible that cytosine-to-uridine (C-to-U) editing events could create new miRNA binding sites as is the case with A-to-I editing.

Other RNA modifications have less drastic effects on the mRNA sequence than RNA editing, but could affect miRNA regulation through other mechanisms, such as altering mRNA structure to reveal or mask miRNA binding sites. For instance, m6A destabilises RNA duplexes, but stabilises RNA structure in the single-stranded context, and can act as a conformational switch [68]. Interestingly, Meyer et al. found that m6A accumulates within the 3′ UTR, and that there is significant overlap between 3′ UTRs having m6A sites and miRNA binding sites [69]. When both m6A sites and miRNA binding sites co-occur within the same 3′ UTR, m6A is located in front of the miRNA binding site 62% of the time. However, this study found that the exact locations of m6A peaks and miRNA binding sites are negatively correlated, with the former tending to cluster near the stop codon and the latter towards the 3′ terminus of the mRNA. Given the important role of m6A in regulating structure and function in other classes of RNA, it is plausible that an m6A-induced change in the structural state of the 3′ UTR alters miRNA targeting. Whether this has a permissive or inhibitory effect on miRNA-mediated RNA degradation remains to be determined, and may differ between mRNAs.

#### 2.1.6. MicroRNAs Can Act as Guides for mRNA Modification

An exciting new possibility for miRNA function was demonstrated by Chen et al., who found that miRNAs can act as guides to specify the location of m6A modification on mRNA in several mouse cell lines [70]. In this study, overexpression of randomly selected miRNAs with sequences overlapping with mRNA m6A sites caused the level of m6A at the corresponding sites to increase, while artificial expression of mutant miRNAs drove de novo accumulation of m6A on mRNAs targeted by these artificial miRNAs. RNA immunoprecipitation experiments showed that manipulating the expression of specific miRNAs altered the frequency of interaction between their mRNA targets and the m6A methyltransferase Mettl3. This is consistent with the findings of Meyer et al. who reported that the target transcripts of the 25 most abundant miRNAs in the brain are significantly more likely to contain m6A, suggesting that miRNA levels may influence the m6A methylation of their targets [69]. These results are compelling, and the possibility that miRNAs directly guide epitranscriptomic modification of mRNAs warrants further investigation.

### 2.2. Piwi-Interacting RNAs

Piwi-interacting RNAs are a class of small regulatory RNAs defined by their interaction with Piwi-like proteins, which are a subset of the Argonaute family. Characteristic features of piRNAs include a length of 26–31 nucleotides (in vertebrates), occurrence in genomic clusters, a bias towards uracil as the first base, and 2′-*O*-methylation of the terminal nucleotide [71]. The primary function of the Piwi pathway in most animals is the control of retrotransposons, which are normally silenced but can become transiently expressed when chromatin relaxes to permit DNA replication, repair, or widespread chromatin remodelling such as that which occurs in the early embryo or during gametogenesis. Piwi proteins and piRNAs are therefore expressed at high levels in stem cell and germ cell lineages of diverse species, as well as in many cancers, and transiently during DNA repair or temporary periods of cell division.

#### 2.2.1. Biogenesis and Mechanisms of Piwi-Interacting RNAs

The biogenesis and maturation of piRNAs has been thoroughly reviewed by Iwasaki et al. [71]. Briefly, piRNAs primarily arise from genomic clusters, the majority of which correspond to transposon sequences; in mammals, they are often transcribed bi-directionally [71]. Although the initial processing of piRNA cluster transcripts has not been fully characterised, it is independent of both Drosha and Dicer [72,73]. Primary piRNA biogenesis from piRNA cluster transcripts produces small RNAs with a 5′-uridine bias which are generally antisense to their targets. Secondary piRNA biogenesis occurs when primary piRNAs guide a Piwi protein to a complementary transcript, which is cleaved by the Piwi protein at a position that is 10 nucleotides downstream of the 5′ end of the loaded piRNA (resulting in a 10th-position adenosine bias within the secondary piRNA population). In this way, transcripts targeted by piRNAs are turned into more piRNAs in an amplifying process referred to as the “ping pong cycle”. The maturation of both primary and secondary piRNAs requires many additional enzymatic steps, including 3′ end trimming. It is noteworthy that piRNAs are characteristically 2′-*O*-methylated on the 3′ terminal base; this modification is applied by the enzyme HENMT1 [74,75,76] and appears to improve piRNA stability, as well as being involved in specific recognition of piRNAs by Piwi proteins [77,78,79,80].

In addition to the degradation of piRNA-complementary transcripts by the endonuclease activity of Piwi proteins, piRNAs can modulate gene expression through epigenetic activation and epigenetic silencing. Although most steps of piRNA production are cytoplasmic, Piwi proteins can re-enter the nucleus and are guided by piRNAs to complementary regions of genomic DNA. In the vast majority of cases, this results in epigenetic silencing by recruitment of chromatin modifiers (reviewed by Iwasaki et al. [71]). However, there are several examples of epigenetic activation by the Piwi pathway. For instance, Piwi can promote euchromatin maintenance in specific cases in *Drosophila* [81]. The Piwi pathway has also been implicated in DNA repair through a similar mechanism; PIWIL2, which is normally silenced in mouse fibroblasts, is transiently upregulated in response to ultraviolet (UV)-induced DNA damage and triggers histone acetylation, chromatin relaxation and subsequent DNA repair [82]. PIWIL2 is also required for chromatin relaxation in response to DNA damage induced by cisplatin, again through a histone acetylation-related mechanism [83].

#### 2.2.2. The Piwi Pathway in the Developing Brain

There is a growing recognition that the Piwi pathway is involved in cell fate decisions during development and regeneration [84]. *Piwil1* in particular has several known functions in neuronal precursors which affect their migration and differentiation. Knockdown of *Piwil1* in newborn cortical neurons in the developing mouse brain produces a defect in neuronal polarisation and radial migration, partly through regulation of microtubule-associated proteins [85]. In a *Piwil1* knockout mouse model, neuronal precursors show increased cell cycle re-entry and delayed differentiation, and neuronal migration and neocortical architecture are aberrant [86]. Another study found that *Piwil2* knockout mice are hyperactive, with altered anxiety-like behaviour; this phenotype is likely neurodevelopmental as the absence of *Piwil2* is present from conception [87]. These findings are consistent with the discovery that de novo mutations in Piwi-like genes are strongly associated with autism spectrum disorders [88], which are increasingly understood to result from abnormal organisation of cortical neurons. To date, the role of individual piRNAs in these neurodevelopmental processes has not been investigated.

#### 2.2.3. Piwi-Interacting RNAs in Differentiated Neurons

In 2012, Rajasethupathy et al. reported that neurons of the sea slug *Aplysia californica* express the only Piwi-like protein found in this species, and several dozen piRNAs [89]. They found that knockdown of PIWI or of one specific piRNA in neuronal cultures impairs long-term facilitation, providing evidence that the Piwi pathway may be involved in learning. In this study, detailed biochemical validation was performed (including confirmation that the putative piRNAs co-immunoprecipitate with PIWI, are depleted by knockdown of PIWI, and are 2′-*O*-methylated at their 3′ termini) to demonstrate that the small RNAs detected were bona fide piRNAs. 

Other authors have reported that the Piwi pathway occurs in mammalian neurons. Lee et al. detected expression of *Piwil1* in cultured mouse neurons and reported that depletion of a specific putative piRNA alters dendritic spine morphology [90]. Other authors have reported dysregulation of piRNAs in response to various manipulations or disease states in rodent and human brain tissue. Putative piRNAs were found to be dysregulated in the rat brain by induced stroke [91], in the human brain by Alzheimer’s disease [92], and in the mouse brain by knockout of *MeCP2* to model Rett syndrome [93]. These studies classified small RNAs as piRNAs based on their size, and on their annotation as piRNAs in one of several online databases. However, most of them did not include 3′ end analysis or demonstrate a functional association of putative piRNAs with Piwi-like proteins through immunoprecipitation, and bioinformatic analysis either did not detect the expected features of piRNAs (such as 1U bias and genomic clustering) or this information was not reported. A recent re-analysis found that piRNA databases contain a subset of sequences which are annotated as piRNAs, but which are actually piRNA-sized fragments of ubiquitously expressed non-coding RNAs such as ribosomal RNAs, transfer RNAs and small nucleolar RNAs [94]. The majority of piRNAs reported from mammalian brain appear to fall into this category. This observation does not rule out the possibility that some bona fide piRNAs are derived from larger non-coding RNAs, and that some of the small RNAs described may be functioning as piRNAs; for instance, a snoRNA-derived piRNA has been confirmed to co-immunoprecipitate with PIWIL1 and PIWIL4 and has an epigenetic activation role in human cancer cells [95]. However, it is likely that the scale of piRNA expression and disease-associated dysregulation in the mammalian brain has been overestimated.

Despite this setback, two recent studies have profiled piRNA expression in the mouse brain using stringent bioinformatic criteria to classify small RNAs as piRNAs. Ghosheh et al. sequenced small RNAs in the size range of piRNAs from the mouse brain [96]. The authors focused on reads which mapped within known piRNA clusters, and found that 7% of reads from the adult brain were derived from piRNA clusters; this subset of reads demonstrated the 1U bias characteristic of primary piRNAs. They also predicted targets for the piRNAs that they identified and reported 41 potential target mRNAs, but over 7500 potential targets within repeat elements, including around 3000 targets on short interspersed elements (SINEs) and 450 on long interspersed elements (LINEs). Although this study did not include biochemical validation of piRNAs, the major bioinformatic signature of piRNAs (including clustering, 1U bias, and targeting predominantly within repetitive elements) was convincingly detected from the adult (but not the juvenile) brain. Nandi et al. sequenced small RNAs from primary mouse hippocampal neurons and classified a population of 5′ U, retrotransposon-derived small RNAs as potential primary piRNAs; they also detected a small number of LINE1 and SINE-associated, 10th-position adenosine small RNAs which may be secondary piRNAs [87]. In addition, they examined *Piwil2* knockout mice and found that LINE1 promoters were broadly hypomethylated in the hippocampus and prefrontal cortex, suggesting a defect in transposon control. This mouse line also showed hyperactivity and reduced anxiety, which implies a functional role for neuronal piRNAs in regulating behavioural responses to novelty.

Other studies have implicated the Piwi pathway in gene regulation in adult neurons without directly examining piRNAs. Earlier work from our laboratory detected low-level expression of *Piwil1* and *Piwil2* in cultured mouse neurons; both genes are dynamically upregulated by neuronal stimulation, and they promote activity-dependent chromatin relaxation at the *Ppp3r1* locus which is required for binding of the regulatory factor ING1 and subsequent expression of *Ppp3r1* [97]. We have also detected expression of all three mouse Piwi-like genes in the adult hippocampus, and have shown that simultaneous knockdown of *Piwil1* and *Piwil2* in the hippocampus enhances contextual fear memory without affecting generalised anxiety [98].

### 2.3. Endogenous Small Interfering RNAs

Endogenous small interfering RNAs, known as endo-siRNAs, are 20–24 nucleotide small RNAs which participate in RNA interference. Endogenous small interfering RNAs arise from long double-stranded RNA (dsRNA) precursors, and the endo-siRNA system likely evolved as an immune response to undesirable dsRNAs such as viral genomes and transposon transcripts [99]. However, long dsRNAs also form when separately transcribed RNAs anneal to each other (as may occur when sense and antisense RNAs are transcribed from the same locus) or when individual transcripts contain large hairpin structures. The production of endo-siRNAs from these dsRNAs likely represents a re-purposing of this pathway for endogenous gene regulation. The cleavage of dsRNAs to form endo-siRNAs is performed by DICER, but does not require DROSHA or other components of the miRNA biogenesis machinery [99]. Another important difference between miRNAs and small interfering RNAs (siRNAs) is that siRNAs bind perfectly to their targets, and it has been suggested that siRNAs may therefore achieve equivalent gene silencing to miRNAs when present at lower levels [100].

#### Are Endogenous Small Interfering RNAs Present in the Brain?

The existence of endo-siRNAs in the mammalian brain remains controversial, partly because these small RNAs are difficult to unambiguously identify. They overlap in size with miRNAs and possibly with piRNAs; they can also be associated with transposons and repetitive elements, and in some species are characteristically 2′-*O*-methylated at their 3′ termini. This means that distinguishing them from both miRNAs and piRNAs is best done by examining the genomic context of putative endo-siRNAs to look for hallmarks of dsRNA formation and processing, such as the bioinformatic prediction of a large hairpin domain or the confirmed generation of sense and antisense transcripts at a potential endo-siRNA producing locus. Smalheiser et al. identified 20–23 nucleotide small RNA sequencing reads from mouse hippocampus that map within eight genes which contain intronic inverted repeats forming large hairpin structures that do not include any annotated miRNAs [101]. These small RNAs are upregulated in samples from mice trained in an olfactory learning paradigm, and are immunoprecipitated by an antibody that recognises all mouse eIF2c (Argonaute) isoforms (but not Piwi-like proteins.) Similar small RNAs were found at the *Ctnna2* locus, where another gene, *Lrrtm1*, occurs within an intron and is transcribed from the opposite direction; these small RNAs probably represent endo-siRNAs derived from annealing of complementary RNAs [101]. Ling et al. also described the formation of dsRNAs from annealing of *Sox4* mRNA and natural antisense transcripts produced from the *Sox4* locus; a specific 24 nucleotide small RNA with Dicer-dependent origin was found to originate from these dsRNAs and has a potential role in the regulation of neurogenesis [102]. Importantly, these papers only considered small RNAs derived from protein coding genes. Conversely, Nandi et al. reported putative 20–24 nucleotide endo-siRNAs of both gene-derived and repeat-derived origin from primary mouse hippocampal neurons [87].

To date, although putative endo-siRNAs have been described in the mammalian brain and are plausibly predicted to have a role in synaptic plasticity, there is no convincing evidence that they directly regulate gene expression or that they are functional components of gene regulatory networks at the synapse. Further experiments (such as specific deletion of siRNA-generating intronic inverted repeats) need to be performed in order to determine whether endo-siRNAs are important for synaptic function or for the development or function of the brain more broadly.

### 2.4. Transfer RNAs

Transfer RNAs are the molecular adaptors which recognise the sequence of mRNA and deliver the corresponding amino acid into the active site of the ribosome for incorporation into the nascent polypeptide chain. They range in size ~75–90 nucleotides and form a universally conserved “cloverleaf” shape, with three loops surrounding a central stem. The 3′ terminus of the tRNA molecule is the attachment site for an amino acid; structurally opposite this site is the anticodon loop, which base pairs with the appropriate mRNA codon to deliver the amino acid to the ribosome. Transfer RNAs have generally been overlooked as potential regulatory molecules and their fragments, when sequenced, have been interpreted as junk produced by the sample extraction and library preparation process. However, the growing recognition that tRNA fragments can have metabolic and regulatory roles is leading to renewed interest in tRNA biology.

#### 2.4.1. Biogenesis and Modification of Transfer RNAs

In eukaryotes, tRNAs are encoded at dozens of loci by both the nuclear and mitochondrial genomes and are transcribed by RNA polymerase III. Newly synthesised tRNA transcripts contain superfluous sequence at both the 3′ and 5′ ends, as well as introns, and undergo several processing steps to remove these non-functional sequences and add the invariant overhanging triplet, CCA, to the 3′ end of the tRNA (reviewed by Phizicky and Hopper [103]). In addition to these changes to the tRNA sequence, extensive post-transcriptional modification occurs. Most eukaryotic tRNAs have over 30 modified bases, including some very large and complex modifications which are not thought to occur on any other type of RNA [103]. Dozens of enzymes are involved in the application of modifications to tRNAs, including some which have no other known function, and some which also modify other RNAs [103].

Following tRNA processing and modification, tRNAs enter a cycle of charging and participation in translation. Charging, or aminoacylation, of tRNAs involves covalent attachment of the correct amino acid to the 3′ terminus of the tRNA by a protein known as an aminoacyl tRNA synthetase (aaRS). One aaRS is associated with each amino acid and this protein recognises all of the tRNAs which carry that amino acid [104]. The function of aminoacyl tRNA synthetases is a major constraint on tRNA sequence, structure and modification; tRNAs which are recognised by the wrong aaRS will misincorporate amino acids into nascent proteins, while tRNAs which are no longer recognised by the cognate aaRS cannot be used by the cell. Aminoacyl tRNA synthetases are part of the quality control pathway for tRNAs, which minimises translation errors caused by damaged tRNAs or incorrect tRNA loading [104]. Interestingly, mutations in genes encoding aminoacyl tRNA synthases produce primarily neurological phenotypes (reviewed by Ognjenović and Simonović [105]), again demonstrating the extraordinary sensitivity of the brain to mutations which disrupt the ability of neurons to precisely regulate protein synthesis.

#### 2.4.2. Regulation of Protein Synthesis by Intact Transfer RNAs

Transfer RNAs are often thought of as housekeeping genes, which are required by all cells in relatively constant quantity in order to carry out basic metabolic processes. However, there are several domains of tRNA function through which intact tRNAs affect the rate or fidelity of protein synthesis, both through established regulatory functions, and in cases where tRNAs or their cofactors are mutated.

There are 20 universal proteinogenic amino acids, but 64 triplet codons, of which 3 are stops and 61 specify an amino acid. There are fewer than 61 unique tRNAs, because of wobble base pairing rules which allow the same tRNA to recognise codons that differ at the third position [104]; humans have tRNAs bearing 49 anticodon sequences [106]. However, most tRNAs are encoded at multiple genomic loci, and in humans there are 513 nuclear and 22 mitochondrial tRNA genes, although this number varies between individuals [106]. Many of these tRNAs are isodecoders, which have the same anticodon sequence but differ elsewhere in their sequence and can interact differently with the ribosome [104,107]; isodecoders are differentially expressed between different tissue types, between proliferating and differentiated cells, and in response to stress [108,109]. The occurrence of different tRNA pools in different cell types provides a mechanism to regulate translation: the pairing of specific tRNAs with specific codons can cause transient pausing of the ribosome during translation, which facilitates co-translational protein folding and is required for the correct formation of the tertiary structure of some proteins (reviewed by Zhang and Ignatova [110]). Concomitantly, tissue-specific tRNA expression in humans correlates with the codon usage of tissue-specific proteins, further demonstrating that the composition of the tRNA pool is regulated by the cell according to its translational needs [109,111].

Further examples of the subtle regulatory capacity of tRNAs are provided by cases where tRNA mutations cause disease, generally despite the presence of functional isodecoder tRNAs in the genome. For instance, mutation of a brain-specific tRNA contributes to neurodegeneration in mice due to stalling of the ribosome. Interestingly, the severe neurodegenerative phenotype is only unmasked when the ribosome rescue protein GTPBP2 is also mutated, revealing the potential interplay between variants in tRNAs and protein-coding genes [112]. A similar effect occurs due to a synonymous mutation in *CFTR*, the gene which causes cystic fibrosis when mutated. A synonymous single nucleotide polymorphism (SNP) in this gene causes detrimental changes to the stability of the encoded protein because translation through this SNP requires a low-abundance tRNA, tRNA^Thr^(CGU), that is particularly rare in human bronchial epithelia [113]. Similarly, the mutation that causes Huntington’s disease interacts with tRNA^Gln^(CUG); this tRNA is not rare, but its charged form becomes depleted in cells expressing mutant huntingtin due to translation of the repeat expansion poly(CAG) [114]. An impaired translation rate as a result of this causes ribosome frameshifting, which is worse in the striatum because this brain region expresses tRNA^Gln^(CUG) at a lower level [114]. Kirchner and Ignatova speculated that this may explain why striatal function is affected early in the progression of Huntington’s disease [104,115].

#### 2.4.3. Transfer RNA Modifications Are Essential for Their Function and Regulation

The many post-transcriptional modifications applied to tRNAs strongly influence their folding, structure and properties (discussed extensively by Guy et al. [116]), and the pattern of modification across the entire tRNA pool shifts dynamically and differentially in response to various sources of cellular stress [117]. Modification of tRNAs within the anticodon loop is essential for wobble base pairing, which enables one tRNA to recognise several different codons which specify the same amino acid; for instance, inosine commonly occurs at the first anticodon position and is able to base-pair with either A, C or U at the third codon position [118]. Some modifications are involved in the correct charging of the tRNA [119,120], and in the maintenance of the correct reading frame [121]. Modifications of tRNAs can also determine whether and how they undergo cleavage to form tRNA fragments. The endonuclease angiogenin cleaves several tRNAs at a position within the anticodon loop [122]; this cleavage is blocked by the presence of 5-methylcytosine, which is applied by NSUN2 or DNMT2 [123,124]. Mutations in NSUN2 are associated with microcephaly and intellectual disabilities in mice and humans [125,126], and the accumulation of abnormal 5′ tRNA fragments in *NSun2* knockout mice causes cellular stress responses and neuronal apoptosis [124]. Although the specific functions of many other tRNA modifications are not yet known, mutations in enzymes which apply tRNA modifications produce predominantly neurological phenotypes, implying that the absence of these modifications is more detrimental to the brain than to other tissue types [9,116]. Further research will likely identify other cellular signalling systems similar to that formed by NSUN2 and angiogenin.

#### 2.4.4. Transfer RNA Fragments Are Novel Regulatory RNAs

It is increasingly recognised that fragmentation of tRNAs is a non-random process; specific endonucleases cleave specific tRNAs in response to cellular signals, and in many cases the fragments thus generated are functional signalling or regulatory molecules within the cell. Small non-coding RNAs derived from tRNA precursors or mature tRNAs are collectively referred to as tRNA fragments or tRFs; the term tiRNA (tRNA-derived stress-induced RNA) refers to tRNA halves produced by a single cleavage within the anticodon loop [127,128]. The quantity of tRFs present in the cell can fluctuate while the population of functional tRNAs remains stable [24,129], indicating that tRNA fragmentation is not merely a means to negatively regulate protein synthesis by reducing translational capacity.

Several studies have linked regulated cleavage of mature tRNAs to the cellular stress response and have implicated this process in neurological disease (specifically via the NSun2–angiogenin–tiRNA axis described previously) [124,130]. Some tRFs are able to negatively regulate translation by interfering with assembly of the cap binding complex required for translation initiation, and this is associated with formation of stress granules which participate in downstream stress signalling [131]. A different population of tRFs from embryonic stem cells also negatively regulates translation by targeting the translation initiation complex; biogenesis of these tRFs is specified by pseudouridine and depletion of the pseudouridine synthase PUS7 dysregulates these tRFs and causes increased protein synthesis and impaired differentiation [132]. Another tRF interacts with the mRNA encoding apolipoprotein E receptor 2 (*APOER2*) to reduce expression of this gene [133]. Although this was identified in the context of respiratory syncytial virus infection, *APOER2* is implicated in Alzheimer’s disease [134] and the potential for this gene to be regulated by a tRF may have relevance to this disorder. Positive regulation of gene expression is also a known mechanism for tRFs; a tRF which binds the mRNAs encoding two ribosomal proteins increases their translation to increase ribosome biogenesis [135]. Other tRFs, including many derived from tRNA precursors as well as some derived from mature tRNAs, are processed by DICER [136] and incorporated into Argonaute proteins, and are able to interact with mRNAs [137,138], although this has not been confirmed to occur in the brain.

Strikingly, tRFs have also been implicated in transgenerational inheritance, a phenomenon by which metabolic, and in some cases behavioural, phenotypes are observed in the unexposed offspring of animals which underwent an environmental manipulation prior to mating [5]. The studies implicating tRFs in this phenomenon have focused on transmission through the male line. Transfer RNA fragments are delivered to developing sperm as they pass through the epididymis [139] and are highly abundant in mature sperm [139]. Sharma et al. observed dysregulation of tRFs in mature sperm following exposure of male mice to low-protein diets, and showed that these tRFs are involved in gene regulation in the embryo [140]. Short et al. found that the male offspring of male mice provided with running wheels showed reduced anxiety, and observed differential expression of small non-coding RNAs including tRFs in the sperm of running mice compared to controls which did not exercise [141]. Chen et al. isolated tRFs from the sperm of male mice which had been maintained on a high fat diet and they report that injection of these tRFs into normal mouse oocytes is sufficient to produce a metabolic disorder similar to that observed in the offspring of high-fat-fed fathers produced by in vitro fertilization [142]. This finding, if confirmed, suggests that tRFs are carriers of epigenetic information which produces the transgenerational metabolic phenotype. The apparent ability of tRFs to convey stress-related information from the father to the embryo emphasises their function as bona fide signalling or regulatory molecules, rather than merely being byproducts of the cellular stress response.

### 2.5. Small Nuclear RNAs

Small nuclear RNAs are the RNA components of the spliceosome, a complex ribonucleoprotein machine which catalyses the removal of introns from newly transcribed RNA. Small nuclear RNAs are 95–200 nucleotides long in mammals, and are rich in uridine, leading to their older common name of “U-RNAs”; they are also referred to as spliceosomal RNAs. There are nine spliceosomal RNAs, all of which are highly conserved; most of them are encoded in the mammalian genome in multiple copies, which supports the requirement for their abundant expression as well as shielding cells against inactivating mutations. Most snRNAs are heavily processed and possess a 5′ cap as well as multiple modified nucleotides, including 2′-*O*-methylated and pseudouridylated positions (reviewed by Karijolich and Yu [10]). These modifications support the strong secondary structure and protein-binding capacity of snRNAs in order to allow assembly of small nuclear ribonucleoproteins (snRNPs), the functional subunits of the spliceosome.

In eukaryotes, the majority of newly transcribed mRNAs and long non-coding RNAs contain introns, non-coding sequences which must be removed to generate the mature and functional RNA. Intron removal, or RNA splicing, is carried out by the spliceosome in nuclear compartments called splicing speckles or simply speckles. The spliceosome dynamically assembles on the precursor mRNA (pre-mRNA); removal of the intron requires a series of reactions which are sequentially catalysed by the various snRNPs which comprise the spliceosome, facilitated by base-pairing between the snRNAs and recognition sequences within the introns and exons of the pre-mRNA (reviewed by Hoskins and Moore [143]). The majority of introns are recognised by the major spliceosome, which is common to all eukaryotes and comprises of the U1, U2, U4, U5 and U6 snRNPs as well as some additional proteins [143]. Most eukaryotes also have some introns which use different recognition sequences, known as U12-type introns. These introns are removed by the minor spliceosome, a process referred to as non-canonical splicing [144]. The minor spliceosome is functionally analogous to the major spliceosome, but has different snRNP components, specifically those including the U5, U11, U12, U4atac and U6atac spliceosomal RNAs (reviewed by Turunen et al. [144]). U12-type introns make up less than 0.5% of all introns in humans, and the minor spliceosome represents less than 1% of all spliceosomes in most human cells. U12-type introns are enriched in genes related to RNA transcription and processing, cytoskeleton function, vesicle transport, and voltage-gated ion channel function [145]; these are all pathways of great relevance to neuronal physiology and function.

#### 2.5.1. Small Nuclear RNAs Participate in Alternative Splicing

While splicing is necessary to generate functional RNA from the majority of genes, it can also be a means of gene regulation and the generation of genetic diversity through alternative splicing. This process involves the selective inclusion and exclusion of exons in order to generate proteins with different amino acid sequences. Alternatively-spliced transcripts are enriched in the brain and the regulation of alternative splicing is considered critical for brain development and function [146]. Alternative splicing is thought to be regulated mostly by proteins and long non-coding RNAs, and probably by epitranscriptomic modifications to mRNA precursors [7,146]. However, there is evidence that snRNAs may play a role in alternative splicing in the brain. A recent study found that disruption of the mouse *Rnu2–8* gene (one of the copies of the U2 snRNA) causes cerebellar ataxia and neurodegeneration [6]. Surprisingly, the mutant U2 snRNA was differentially expressed between tissues, with the highest level detected in granule cells of the cerebellum, which were progressively lost as the mice aged. Transcriptome-wide profiling revealed widespread abnormalities of alternative splicing in the cerebellum as a result of this mutation. Further evidence that snRNAs can be expressed in a tissue-type specific manner is provided by a study which found differential expression of different snRNA variants between tissue types and developmental stages of several species [147]. However, the effect of this pattern on the regulation of splicing is currently unknown.

#### 2.5.2. The Minor Spliceosome Is Implicated in Neurological Function

Perturbations of the minor spliceosomal snRNAs result in cellular dysfunction which disproportionately affects the developing nervous system. Removal of U12 introns by the minor spliceosome is substantially slower than removal of U2 introns by the major spliceosome, and this has been proposed as a gene regulatory mechanism [148]. U12 introns are almost absent from housekeeping genes, but are enriched in genes related to DNA and RNA metabolism, cytoskeletal organisation, vesicular transport, and voltage-gated ion channel activity [145,148,149], which are pathways with relevance to neuronal function. This may explain why mutations in minor spliceosomal snRNA genes (all of which are single-copy genes) produce neurological phenotypes. A mutation in the minor spliceosomal snRNA U12 has been reported to cause autosomal recessive cerebellar ataxia and mild intellectual disability with evidence of cerebellar atrophy [150], and defective splicing was observed in RNA isolated from peripheral blood mononuclear cells of the patients in this study. Inactivating mutations of the minor spliceosomal snRNA U4atac cause microcephalic osteodysplastic primordial dwarfism, type I (MOPD-I), a condition characterised by severe microcephaly and intellectual disability with proportionate short stature and skeletal abnormalities [151,152,153,154]. The microcephaly observed in children with MOPD-I implies that neuronal precursors are among the cell types most affected by the loss of this snRNA. Neuronal precursor loss resulting in microcephaly is also observed after deletion of the minor spliceosomal snRNA U11 in the developing mouse cortex [155], and inactivation of U12 in the postnatal mouse retina results in apoptosis of differentiating retinal cells [156]. The observation that all four snRNAs unique to the minor spliceosome are enriched in the developing mouse brain [156] further underscores their importance in neurodevelopment. Finally, mutations in proteins which participate in or interact with the minor spliceosome have also been implicated in brain function; for instance, minor splicing dysfunction is observed with Fused in sarcoma (FUS) mutations associated with the devastating degenerative neuromuscular condition, amyotrophic lateral sclerosis [157].

#### 2.5.3. Small Nuclear RNAs Are Post-Transcriptionally Modified

Small nuclear RNAs contain a number of post-transcriptional modifications, predominantly pseudouridylated and 2′-*O*-methylated positions. The majority of modifications on snRNAs are applied in the Cajal body during snRNA maturation by small Cajal body specific RNAs (discussed later in this review). However, some snRNA modifications are applied in an RNA-independent manner by pseudouridine synthases including Pus7p and Pus1p [158,159]. Several lines of evidence point to their importance for snRNA function, including conservation of modified locations between species, and clustering of modifications in functionally-important regions of the RNA [10]. Specific functions have been identified for a number of modified positions. For instance, a pseudouridine within the U1 snRNA enhances its interaction with the 5′ splice site of a specific RNA [160], while each of four specific 2′-*O*-methylated bases at the 5′ end of U2 snRNA are required for splicing [161]. Loss of modification at many positions has been shown to impair or prevent spliceosome assembly, or to reduce the efficiency of splicing (reviewed by Karijolich and Yu [10]). However, to our knowledge, no individual modifications on snRNAs have been implicated in mediating the influence of cellular processes on the spliceosome. Further research may well uncover fractional or activity-dependent snRNA modifications as mechanisms to regulate splicing, as is the case with several long non-coding RNAs which are involved in alternative splicing [7,8].

### 2.6. Small Nucleolar RNAs

Small nucleolar RNAs and small Cajal-body RNAs (scaRNAs) are nuclear RNAs which are primarily involved in the post-transcriptional modification of other RNA species. There are two subclasses of snoRNAs, which are defined by specific sequence motifs. C/D box snoRNAs direct 2′-*O*-methylation at specific sites of their RNA targets, while H/ACA box snoRNAs guide isomerisation of specific uridine residues in target RNAs to pseudouridine. C/D box and H/ACA box snoRNAs are predominantly localised to the nucleolus, which is the site of ribosome biogenesis, and the vast majority of their target sites occur on the 18S and 28S ribosomal RNAs (reviewed by Henras et al. [11] and Reichow et al. [162]). Conversely, scaRNAs are found in the Cajal body, a small nuclear body which was discovered in neurons but also occurs in other metabolically active cell types. Many scaRNAs are structurally and functionally indistinguishable from snoRNAs, although some can be substantially larger than snoRNAs and possess both C/D and H/ACA motifs [162]. Most scaRNAs with known targets are involved in 2′-*O*-methylation and pseudouridylation of small nuclear RNAs.

#### 2.6.1. C/D Box Small Nucleolar RNAs

C/D box snoRNAs range in size from ~70 to ~160 nucleotides, and are defined by the presence of the C-box (RUGAUGA, R = G or A) and D-box (CUGA) motifs, which occur near the 5′ and 3′ ends of the RNA respectively [163]. Most C/D box snoRNAs also contain internal copies of the C-box and D-box motifs, referred to as the C’ and D’ boxes, which are often less well conserved. The C and D boxes interact to form a “kink-turn” structure which brings the 5′ and 3′ ends of the snoRNA together, and in cases where both the C’ and D’ boxes are present, an additional “kink-loop” structure also forms [164,165,166,167]. This structure is surrounded by the core machinery of the C/D small nucleolar ribonucleoprotein (snoRNP), consisting of four highly conserved proteins, Nop56, Nop58, 15.5k (Snu13) and fibrillarin, the last of which is recognised as the snoRNA-dependent 2′-*O*-methyltransferase [164,168,169,170]. Each C/D box snoRNA has either one or two target sites which base-pair with the target RNA over an 8–20 nucleotide region and direct fibrillarin to the exact site of 2′-*O*-methylation, which is almost always five nucleotides upstream of the D or D’ box [163,164,171]. The majority of C/D box snoRNAs in mammals have known ribosomal RNA (rRNA) targets, but several dozen have unknown functions.

Most C/D box snoRNAs are ubiquitously expressed, consistent with their function in guiding rRNA modifications that are necessary for proper function of the ribosome. However, some C/D box snoRNAs have tissue-specific expression patterns, including several that are expressed predominantly or only in the brain [172,173]. Two of these brain-specific snoRNAs (snord115 and snord113) are differentially regulated in the hippocampus following contextual fear conditioning [174], implying a function in memory consolidation. Preliminary data from our laboratory have also revealed several more C/D box snoRNAs which are dynamically expressed in response to fear-related learning.

To date, the most compelling example of C/D box snoRNAs with neurological functions is given by the contribution of snord115 and snord116 to Prader-Willi syndrome (PWS), a disorder characterised by intellectual disability and neonatal growth deficiency followed by hyperphagia and obesity [175]. Prader-Willi syndrome is caused by the loss of a paternally expressed imprinted domain on human chromosome 15, which includes multiple tandem repeats of both snord115 and snord116, both of which are implicated in the PWS phenotype (reviewed by Cavaille [176]). Snord116 is carried within the minimum critical region which is lost in PWS, and may be the primary genetic cause of this syndrome [177,178,179]; deletion of all copies of this snoRNA in mice produces a neonatal growth defect followed by hyperphagia and behavioural abnormalities in adults [180]. However, to date no RNA target for snord116 has been demonstrated and its function is unclear. Conversely, the target RNA of snord115 has been identified: it does not interact with rRNA, but instead targets a region in exon V of the serotonin receptor *Htr2c*. This gene is essential for the regulation of appetite and feeding behaviour in humans and animals [181,182,183]. The interaction between snord115 and *Htr2c* mRNA inhibits an alternative splicing event which drives the expression of a truncated isoform of *Htr2c* [183]. The *Htr2c* transcript also undergoes ADAR2-mediated A-to-I RNA editing which alters the amino acid sequence of the encoded protein; the presumed 2′-*O*-methylation of *Htr2c* mRNA by snord115 blocks editing by ADAR2 and promotes expression of the unedited protein sequence [184]. Through both effects, snord115 promotes production of the fully-functional 5HT2C protein as opposed to less-functional isoforms. Therefore, loss of snord115 contributes to appetite dysregulation in mice [185,186]. Snord115 expression is lost in most PWS patients [176], and dysregulation of *HTR2C* is expected to contribute to the human PWS phenotype in most cases; however, loss of snord115 alone is not sufficient to cause PWS in humans [187].

In addition to the established functions of C/D box snoRNAs in the brain, ongoing research is suggesting new ways in which these small RNAs may regulate nervous system function. The expression of some snoRNAs has a daily rhythm in the *Drosophila* brain [188], and deletion of snord116 in mice causes profound disruption of circadian DNA methylation, with expected patterns of DNA methylation perturbed at 97% of diurnally rhythmic CpG sites in the cortex [189]. This may be related to previously observed sleep dysregulation in the snord116-lacking mouse [190] as well as in people with PWS [175,190]. The role of C/D box snoRNAs in mRNA splicing may also be much broader than is currently appreciated. A recent study found that snord27 (which has a known rRNA target) is involved in alternative splicing of the transcription factor E2F7 pre-mRNA, and knockdown of this snoRNA affects splicing of several other mRNAs through base complementarity with the entire snoRNA sequence, not just the canonical targeting region [191]. Another study found that many orphan human snoRNAs have bioinformatically predicted target sites near alternative splicing junctions [192]. Alternative splicing is more widespread in the nervous system than in most other tissue types and is dynamically regulated in ways that are critical for neurodevelopment and plasticity [146], making this non-canonical snoRNA function particularly interesting in the context of neurobiology. Snord3a has been implicated in prion disease; it is overexpressed in the blood of humans with Creutzfeld-Jakob disease of genetic origin, and in the brains of transgenic mice with an equivalent mutation, with the overexpression increasing in magnitude as the disease progresses [193]. Finally, several recent publications have suggested that C/D box snoRNAs can be processed into smaller regulatory RNAs [137,194]. Given that C/D box snoRNAs are known to form non-methylating ribonucleoproteins (RNPs) [191] and 76 human C/D box snoRNAs do not have identified rRNA target sites [195], continued investigation of their alternative functions is likely to reveal more ways in which these RNAs have been repurposed to support neurodevelopment and plasticity.

#### 2.6.2. H/ACA Box Small Nucleolar RNAs

H/ACA box snoRNAs are approximately 150–300 nucleotides long, and are defined by the H-box (ANANNA, N = any nucleotide) and ACA-box (AYA, Y = C or U) sequence motifs [11,162,195]. The secondary structure of H/ACA box snoRNAs is often described as hairpin-hinge-hairpin-tail; each hairpin consists of 60–75 nucleotides and includes a structure known as a pseudouridylation pocket, where two parts of a discontinuous targeting sequence are brought together by the folding of the snoRNA to create the active site which interacts with the target RNA to specify the location of pseudouridylation [196]. The H-box motif occurs in the hinge region between the two hairpins, while the ACA-box is located in the tail. H/ACA box snoRNAs form a snoRNP with four core proteins: GAR1, NHP2, NOP10 and dyskerin, the last of which is the snoRNA-dependent pseudouridine synthase [196,197]. One H/ACA box snoRNA is known to be specifically expressed in the brain; snora35 is encoded within an intron of *HTR2C* [173]. To date, there is little evidence to imply neuronal functions for specific H/ACA box snoRNAs; however, ongoing efforts to profile dynamic pseudouridylation in neurons may yet implicate these small RNAs in neural plasticity.

#### 2.6.3. Small Cajal Body RNAs

Small Cajal body-specific RNAs are structurally and functionally indistinguishable from snoRNAs, but are defined by their localisation to the Cajal body. Unlike the nucleolus, the Cajal body is not involved in ribosome biogenesis, and lacks ribosomal DNA (rDNA) domains and RNA polymerase I machinery. Concomitantly, scaRNAs target 2′-*O*-methylation and pseudouridylation onto non-rRNA targets, most of which are small nuclear RNAs, consistent with the Cajal body’s role in the final stages of spliceosomal RNA maturation [198]. Small Cajal body-specific RNAs can be differentiated from snoRNAs by the targeting sequences which direct them to the Cajal body. C/D box scaRNAs have UG dinucleotide repeats [199], whereas H/ACA box scaRNAs contain CAB boxes (UGAG) located in the loops of one or both hairpin structures [200,201]. CAB boxes are also a feature of hybrid scaRNAs which are much larger than other snoRNAs and scaRNAs and contain both C/D box and H/ACA box domains. Several such scaRNAs have been immunoprecipitated by both fibrillarin and dyskerin [202], indicating that they simultaneously function as both 2′-*O*-methylation and pseudouridylation guides. 

To date, scaRNAs with brain specificity or neuron-specific functions have not been reported. However, the role of scaRNAs in the maturation of the splicing machinery suggests that neurons could be among the cell types most sensitive to disruption of scaRNA activity, given that alternative splicing is particularly prevalent in the brain. This hypothesis is supported by observations that decreased expression of 12 scaRNAs in congenitally abnormal human hearts is associated with dysregulated alternative splicing of several genes which regulate heart development [203]; although the authors did not biochemically demonstrate a defect in snRNA modification, the splicing abnormalities observed strongly suggest that spliceosome function is perturbed.

Characterisation of scaRNAs is also complicated by the mis-annotation of many scaRNAs as either C/D box or H/ACA box snoRNAs; in many databases, only those scaRNAs with an unusual structure (such as those containing both C/D and H/ACA motifs) are reliably annotated as such. Larger scaRNAs also fall into a size range which is often missed by library preparation for both small and large RNA sequencing, and targeted approaches such as RNA capture-seq may be necessary to better understand their function.

#### 2.6.4. Emerging Domains of Small Nucleolar RNA and Small Cajal Body-Specific RNA Function

The primary functions of snoRNAs and scaRNAs as guides for post-transcriptional RNA modification have been well established. However, many questions remain about the potential regulatory and adaptive functions of these modifications, as well as the growing list of non-canonical functions of snoRNAs and scaRNAs. Recent improvements have been made to the sensitivity and accuracy of biochemical profiling methods for both 2′-*O*-methylation [204] and pseudouridine [205], resulting in a wealth of new data that raise questions about snoRNA and scaRNA function.

The occurrence of 2′-*O*-methylation and pseudouridine on rRNA is universally conserved; humans share the core machinery of both the C/D and H/ACA snoRNPs with bacteria and archaea [11]. The specific modification sites within rRNAs are also highly conserved, and cluster within the rRNA regions most important for function of the ribosome [11,206]. Genetic deletion of either fibrillarin or dyskerin produces profound growth defects in yeast [168,207,208] and deletion of fibrillarin is lethal in mammals at the early embryonic stage [209], providing further evidence that the canonical function of snoRNAs is indispensable for normal ribosome function and protein synthesis. However, only a few specific snoRNAs and their catalysed modifications have been individually linked to negative phenotypes. It has also recently been shown that eukaryotic rRNAs contain several fractionally modified positions, which bear a modification in some, but not all, mature ribosomes [11,210,211]. Several authors have speculated that ribosome heterogeneity—the regulated and intentional production of ribosomes with some expected positions left unmodified—may be a mechanism by which cells control translation rate, translation fidelity, or selection of mRNAs for translation [210,211,212].

Another emerging possibility is that some snoRNAs or scaRNAs modify mRNAs. Some mRNAs are detectable in fibrillarin immunoprecipitates from HEK293T cells, indicating association of mRNA with the C/D box snoRNA machinery [202]. A recent study found low-level 2′-*O*-methylation of some mRNAs from HEK cells and a non-random distribution pattern for this mark, which appears to occur preferentially near splicing junctions [204]. This implies that 2′-*O*-methylation of mRNA could affect splice site selection. There is evidence that 2′-*O*-methylation of mRNA attenuates the translation rate by affecting the interaction between the mRNA and tRNA [12]. It is also plausible that 2′-*O*-methylation of mRNA may affect its structure or stability, as is the case for rRNA, although there is currently no evidence to support this idea. Pseudouridine also occurs in eukaryotic mRNAs and long non-coding RNAs, and in the brain, it responds dynamically to stress [205,213]. While the majority of confirmed pseudouridine sites in mRNA are created by snoRNA-independent pseudouridine synthases, mRNAs are found in dyskerin immunoprecipitate [202]. Pseudouridylation of mRNA dramatically affects its function and translation, and the possibility of snoRNA-guided mRNA pseudouridylation is another dimension through which these small RNAs may participate in coordinating gene regulation.

Finally, it is likely that at least some snoRNAs are processed into smaller fragments which have regulatory roles. Sequencing of small RNAs from various mammalian tissues, including brain, reliably identifies a large population of snoRNA fragments in the 20–30 nucleotide size range [137,214]. Mouse embryonic stem cells lacking *DICER* and *DGCR8* show altered snoRNA fragment profiles [214], and there are many reports of snoRNA-derived miRNAs including some which have been confirmed by immunoprecipitation to interact with Argonaute proteins [194,215,216]. There are also reports of snoRNA-derived piRNAs which co-immunoprecipitate with Piwi proteins in mammalian cell lines [95,217]. Together, these findings indicate that at least some snoRNA fragments are functionally associated with the RNA-interference machinery and can influence gene expression independently of the function of the parent snoRNA.

## 3. Emerging Technologies

### 3.1. Profiling of RNA Modifications with Single Base Resolution 

Our understanding of many RNA modifications is hampered by the lack of technologies to detect them. Recently developed methods to profile some epitranscriptomic marks (including m6A, m5C, pseudouridine, and 2′-*O*-methylation) at single-base resolution with high throughput has enabled unprecedented understanding of where, when and how these marks accumulate on small and large RNAs [204,205,218,219]. As techniques become available to profile more modifications in this way, we will gain greater insight into the epitranscriptome as a whole, including the interactions between different modified RNA species.

### 3.2. Site-Directed Manipulation of the Epitranscriptome with CRISPR-Cas13 

The RNA-guided RNase Cas13, and its RNase-deficient derivatives, can be engineered to modify RNAs with single-base precision. A fusion protein consisting of Cas13b and the adenosine deaminase domain of ADAR2 was used to drive A-to-I editing site-specifically while avoiding the sequence constraints of the wild-type enzymes [220]. We predict that additional fusion proteins between Cas13b and the catalytic domains of epitranscriptomic reader and eraser proteins will become available, enabling site-directed manipulation of RNA modifications for both investigative and therapeutic purposes. 

### 3.3. Tools for Examining RNA Trafficking and Localisation in Neurons 

There is a growing understanding that RNA trafficking and localisation are essential for neuronal function, where rapid and regulated protein synthesis may need to occur as far as one metre from the cell nucleus. New technologies are emerging to interrogate specific RNA molecules and RNA functionalisation with greater temporal and spatial resolution than ever before. Approaches such as bio-orthogonal labelling of nascent RNA using tagged nucleobase analogues [221] and spatially restricted chemical tagging of RNA [222] will enable improved tracking, visualisation and capture of RNA molecules from neuronal sub-compartments under activity-dependent conditions, and several techniques to profile the secondary structure of RNA have recently been published [223,224,225]. This will permit research into the activity-dependent functionalisation of RNA molecules which is not possible with current technology.

## 4. Outlook

The majority of small RNA biotypes are represented in the mammalian brain, where they are an integral component of regulatory processes which underlie most nervous system functions (Table 1). We are beginning to recognise the contribution of RNA modifications to these regulatory processes, and ongoing research will continue to uncover the contributions of small RNAs to the neuronal epitranscriptome.

Epitranscriptomics is a discipline in its infancy. Hundreds of RNA modifications have been confirmed by mass spectrometry to exist in mammalian RNA [226], including 24 recently identified from small RNAs of 16–28 nucleotides in size [227]. However, profiling methods with single-base resolution are only available for a handful [226] and have mostly been applied to cell lines at this stage. Most single-base profiling methods are unable to detect modifications occurring on small RNAs, and many require quantities of input RNA which are not feasible when studying neurological processes in vivo. Further refinement of these methods, and the development of techniques to detect RNA modifications which currently cannot be profiled, should provide new insights into modification of small RNAs, as well as the interactions between small RNAs and other modified positions. Small RNA activity and RNA modification are also tightly regulated both temporally and spatially, and emerging technologies which improve spatial and temporal resolution and cell type specificity for biomolecular profiling of neurons are likely to enhance our ability to identify functionally relevant small RNA expression and RNA modification which are currently not detectable.

Another lesson from the research described in this review is the potential functional relevance of small RNAs that are derived from larger RNA species, and which have typically been dismissed as degradation products arising from sample preparation and sequencing. Most small RNA sequencing analysis also rejects sequences which do not perfectly match the reference genome, which may mean that reads corresponding to modified or edited small RNAs are discarded at an early stage of data analysis and not considered further. Finally, a recent analysis has highlighted issues with small RNA classification in online databases [94] and provided an example of how automated small RNA annotation based on databases may result in substantial misinterpretation of results. Together, these observations highlight the importance of maximising RNA quality and paying careful attention to data analysis to maximise the opportunity to discover and characterise functional small RNAs.

In this review, we have discussed the functions of several classes of small non-coding RNA in the brain, and explored their roles as guides, recipients, and interacting partners for dynamic RNA modifications. The ability of RNA modification to immediately and dramatically affect RNA folding, base-pairing and stability functionalises RNA molecules to rapidly integrate information from changing environmental inputs. We have presented cases of the interplay between small RNAs and the epitranscriptome controlling state-dependent functions of neurons, as well as examples of regulatory mechanisms from other cell types. We expect that ongoing research will discover more ways in which this relationship increases the complexity of information processing in the brain, with particular relevance to higher-order processes such as learning and memory.

## Figures and Tables

**Table 1:** Classes of small RNA occurring in the mammalian brain.

Name	Abbreviation	Features
MicroRNA	miRNA	Length ~19–22 nt, associated with Argonaute proteins, reduces gene expression through partial complementarity with the 3′ UTR of mRNA. Produced from hairpin precursors by Drosha and Dicer. Processing of precursors modulated by base modifications. Mature miRNAs subject to RNA editing, base modification and 3′ tailing. Interactions with mRNA can be modulated by mRNA modification. May be involved in guidance of m6A on mRNA. Existence in neurons well established, with many roles in regulation of brain functions.
Piwi-interacting RNA	piRNA	Length ~24–31 nt, associated with Piwi-like proteins, reduces transposon expression through transcript degradation and epigenetic silencing. Also represses some mRNAs, and has epigenetic activation roles in some contexts. Characteristically 2′-*O*-methylated on the terminal base; this modification is stabilising and involved in specific recognition by Piwi proteins. Existence in neurons well established, although misclassifications have occurred. Small but growing body of evidence showing involvement in neuronal gene regulation.
Small interfering RNA	siRNA	Length ~20–24 nt, associated with Argonaute proteins, reduces gene, virus and transposon expression through perfect complementarity with any region of target RNA. Produced from dsRNA precursors by Dicer without requirement for Drosha. Existence in neurons controversial; likely confined to retrotransposons and a small number of genes with intronic hairpin elements.
Transfer RNA	tRNA	Length ~75–90 nt, molecular adaptors which recognise mRNA codons and transfer the corresponding amino acid to the ribosome during translation. Hundreds of variants with small sequence differences. Carry dozens of modified nucleotides which affect stability, interactions with cofactors, and mRNA decoding. Some modifications regulate enzymatic cleavage of tRNAs.
Transfer RNA fragments	tRF	Length ~20–50 nt, various fragments of tRNAs which are produced through non-random processes and have regulatory and signalling roles within the cell, particularly relating to stress. Generally, they have the same modified nucleotides that were present on the parent tRNA. Implicated in transgenerational inheritance of behavioural and metabolic phenotypes after dietary and environmental manipulation in mice.
Small nuclear RNA	snRNA	Length ~95–200 nt, components of the spliceosome, the ribonucleoprotein machine which splices introns out of pre-mRNA. Heavily modified, mostly with 2′-*O*-methylation and pseudouridine, which are required for RNP assembly and normal splicing. Small nuclear RNAs of the minor spliceosome may regulate alternative splicing and cause severe brain abnormalities when mutated.
Small nucleolar RNA	C/D box snoRNA	Length ~70–160 nt, occur in nucleolus, specify the location of 2′-*O*-methylation applied to target RNAs (primarily rRNA) by the methyltransferase fibrillarin. Family contains many “orphan” RNAs without known target. Several occur within Prader-Willi syndrome locus, involved in the regulation of serotonin receptor, circadian rhythm, and alternative splicing.
Small nucleolar RNA	H/ACA box snoRNA	Length ~150–300 nt, occur in nucleolus, specify the location of pseudouridylation of target RNAs (primarily rRNA) by dyskerin. Family contains many “orphan” RNAs. Currently little evidence to imply a role in brain function.
Small Cajal-body RNA	scaRNA	Length ~70–400 nt, occur in Cajal body. Structurally and functionally indistinguishable from snoRNAs but have many more possible structural variants, including the possibility of carrying both C/D and H/ACA motifs. Specify the location of pseudouridine, 2′-*O*-methylation or both on target RNAs (primarily snRNAs). Indirectly required for splicing. Not specifically implicated in brain function to date.

Abbreviations: nt, nucleotide; UTR, untranslated region; mRNA, messenger RNA; dsRNA, double stranded RNA; pre-mRNA, messenger RNA precursor; RNP, ribonucleoprotein; rRNA, ribosomal RNA.
